# *Heracleum persicum* Desf. ex Fisch., C.A.Mey. & Avé-Lall. fruit essential oil: content, antimicrobial activity and cytotoxicity against ovarian cancer cell line

**DOI:** 10.1186/s12906-023-03892-2

**Published:** 2023-03-21

**Authors:** Mansureh Ghavam

**Affiliations:** Department of Range and Watershed Management, Faculty of Natural Resources and Earth Sciences, University of Kashan, Kashan, Iran

**Keywords:** Iranian traditional medicine, Essential oil content, Ovarian cancer, Microbial strains, *Heracleum persicum*

## Abstract

**Background:**

One of the most important aromatic species of the *Apiaceae* family is *Heracleum persicum* Desf. ex Fisch., C.A.Mey. & Avé-Lall, which used as a spice and condiment in food. It is widely used in traditional Iranian medicine due to its anti-toxic properties. The aim of this study was to evaluate the essential oil of this plant in terms of content and ingredients, cytotoxicity and antimicrobial activity.

**Methods:**

The fruit of *H*. *persicum* was collected in June 2019 from Maragheh region of Kashan, Iran. The essential oil was extracted by water distillation using Clevenger for 3 h. Identification analysis of *H*. *persicum* fruit essential oil (HPFEO) components was performed using gas chromatography-mass spectrometry (GC–MS). Evaluation of the effect of the HPFEO on the growth and proliferation of ovarian cancer cell line (OVCAR-3) was performed using MTT colorimetric method. Its antimicrobial activity was evaluated by agar diffusion method, minimum inhibitory concentration (MIC) and minimum bactericidal/fungal lethality concentration (MBC/MFC).

**Results:**

The results of analysis of the HPFEO by GC–MS showed that there were 35 compounds with 99.54% relative content. Hexyl butyrate (35.24%), octyl 2-methylbutyrate (11.65%), octyl isobutyrate (9.23%), and octyl acetate (8.42%) were the predominant compounds. Examination of cell survival showed that the viability of cells decreased depending on the concentration of the HPFEO in 24 h and had value of IC_50_ ~ 12.08 μg/ml against OVCAR-3 cell line. The strongest inhibitory activity of the HPFEO was against Gram-negative bacteria *Pseudomonas aeruginosa* and *Salmonella paratyphi*-*A serotype* (MIC < 62.50 μg/mL). Also, the strong inhibitory and lethal activity of this essential oil against *Candida albicans* (MIC and MBC 250 μg/mL was one degree weaker compared to nystatin (MIC = 125 μg/mL).

**Conclusions:**

Thus, HPFEO, in addition to its traditional use, may have a strong and potential potential for the production of anti-proliferative and antimicrobial drugs.

## Introduction

For centuries, people around the world have learned how to use plants to fight disease and stay healthy. Existing traditional medicines form the basis of affordable health care and are an essential source of livelihood for indigenous and rural populations [1]. From time immemorial, medicinal plants have been a popular source of bioactive substances used in traditional therapies and have been a repository for modern therapies in modern medicine [2].

Essential oils are one of the most important secondary metabolites in medicinal plants, spices and especially aromatic, which are commonly used as flavoring agents in food products, beverages, perfumes, medicines and cosmetics. Analysis of phytochemical compounds of essential oils has led to the identification of their components and the increasing use of these compounds, especially in the cosmetics and pharmaceutical industries, has increased the demand for their use [3].

Essential oils have shown a wide range of antimicrobial activity against human pathogens, both plankton and immobilized [4–6] The antimicrobial potential of essential oil has long been known and in-depth data on the components responsible for this activity and how it works are available [7]. In fact, essential oils extracted from plants contain various antimicrobial compounds, such as aldehydes, alcohols, phenols, ketones, esters, and terpene hydrocarbons [8].

Some essential oils are promising anti-cancer drugs and are currently being investigated for their cytotoxic and anti-proliferative activities in cancer cell lines or experimental animals. Various mechanisms have been reported for the cytotoxic effects of essential oils or their compounds. These include induction of cell death by apoptosis and/or necrosis, anti-mutation, anti-proliferative, antioxidant, cell cycle arrest, and loss of major organ function [9]. Studies show that specific components of essential oils increase the cytotoxic activity of chemotherapeutic drugs (dutaxel, paclitaxel, 5-fluorouracil) in different cell lines and thus allow their dose to be reduced with the same effect [10, 11].

The *Apiaceae* family is one of the largest plant families with 438 genera and 3,500 species that are mostly distributed in the northern hemisphere [12]. This genus has 131 genera and 365 species in Iran, of which 118 species are exclusive to Iran [13]. The genus *Heracleum* has 10 species, 4 of which are native to Iran. One of the most important native species of *Heracleum persicum* Desf. ex Fisch., C.A.Mey. & Avé-Lall is the Persian name for “Angdan” and “Golpar” [14]. It is an annual, fragrant herbaceous plant [15] that is widely distributed in Iran, but has the best growth in humid areas, especially in the mountainous areas of northern Iran at an altitude of more than 1500 m above sea level [16]. fruit and stem of this plant are used locally as a spice and additive in various pickles [17], and young plant stems are used to prepare pickles [18]. In traditional medicine is used as an anti-worm, anti-flatulence, appetizer, disinfectant, diuretic, and analgesic [19–22]. Various biological properties including antioxidant activity, antitumor, insecticide, antimicrobial, anti-inflammatory, anti-cancer, anticoagulant, anti-diabetic, and analgesic have been reported for this plant [17].

Traditionally, the root and fruit of this plant are used to treat epilepsy in Iran [23]. It is widely used in traditional Iranian medicine due to its anti-toxic properties. In Iran, the people of Ahar city of East Azerbaijan use from the seeds and leaves of this plant to remove heartburn [24]. The people of northeastern Khuzestan use from its fruit and leaves to treat heartburn and heartburn and diarrhea, anticonvulsants and pain, easy and soothing digestion [25] and the people of Lorestan from its leaves and seeds as a spice and food in internal use as an antimicrobial, heart tonic, anti-flatulence, diaphoretic, diuretic, excretion of intestinal parasites, memory enhancement, and as an external use for the treatment of pimples, abscesses, boils [26]. The people of Mahneshan city of Zanjan province use for stomach and diarrhea, treatment of diarrhea, digestion [27]. The people of Mashhad use fruit to treat hiccups, appetite suppressants, flavoring, windbreak, and stomach tonic [28]. This plant has a penetrating odor and its smell can stop hiccups. Its juice is used for poor memory, forgetfulness, dizziness, and dementia [29]. Decoction of fruits is used as an antitoxin, tonic for the stomach, liver, and kidneys [30].

Various compounds in the HPFEO have been identified and recorded. The predominant and major constituents of HPFEO have been reported in various previous studies and include hexyl ester and octyl ester [31], hexyl butyrate and hexyl hexanoate [32], hexyl butyrate and octyl acetate [33–35], *trans*-carveol and α-terpineol [36], hexyl butanoate and octyl isobutyrate [37], (*E*)-anethole and octyl-2-methyl butanoate [38], and butanoic acid hexyl ester and β-ethyl hexyl acetate [39]. Analgesic and anti-inflammatory [21], antitumor and antibacterial [40], cytotoxic [33, 34, 38], antifungal [31], stimulation of the reproductive system [41], hepatoprotective [42] insecticide [43, 44], and antioxidant and antimicrobial activity [38] of HPFEO have been confirmed.

To the best of our knowledge, no previous studies on the cytotoxic activity of *H*. *persicum* essential oil against intestinal human ovarian cancer cells and antimicrobial activity against many microorganisms such as *Streptococcus pyogenes*, *Staphylococcus epidermidis*, *Shigella dysenteriae, Salmonella paratyphi*-*A serotype* and *Aspergillus brasiliensis* was not performed simultaneously. Therefore, in addition to ethnobotanical study of *H. persicum* in Iran, the present study aimed to evaluate the HPFEO in terms of content and chemical ingredients by GC–MS, cytotoxic activity by MTT, and antimicrobial activity by zone of Inhibition, MIC and MBC/MFC designed and done.

## Materials and methods

### Plant material and preparation of the HPFEO and content determination

The fruit of *H. persicum* was collected in June 2019 from Maragheh region of Kashan, Iran (N 33˚41ʹ24 and E 51˚ 26ʹ 45 ʺ with an altitude of 2086 m above sea level). Permission for collection of plant materials obtained from the Agricultural Jahad Office and also the owner of the farm (Maraghe farmer). The study is in compliance with relevant institutional, national, and international guidelines and legislation.This plant was identified and verified by Mansureh Ghavam and was recorded and kept under code 1110 in the herbarium of the Faculty of Natural Resources and Earth Sciences of the University of Kashan, Kashan, Iran.. The plant material was dried at room temperature (25 °C) in the shade and pulverized by electric mill. 100 g of fruit powder was weighed and the HPFEO was extracted by water distillation using Clevenger for 3 h. The essential oil was separated from the water by sodium sulfate and stored in a dark container at 4 °C. The extraction of HPFEO was repeated three times and the yield was reported as mean ± standard deviation in terms of weight [45].

## Analysis of identification of the HPFEO components

The GC–MS system was equipped with a 30-m HP-5MS column, an internal diameter of 0.25 mm, and a thickness of a static phase layer of 0.25 μm. The analysis was performed using helium gas as a carrier gas with a flow rate of 1.5 ml per minute and an ionization energy of 70 electrons. The following temperature programming was performed: (a) 60 °C for 0 min, b) increase of 3 °C per minute from 60 °C to 246 °C. The temperature of the injector and detector was 250 °C. The linear retention (RI) indices of the components were determined with a series of *n*-alkane homologs under the same operating conditions. The compounds were identified by comparing their RI and mass spectra with spectral assemblies in the NIST library (NIST-14). The relative abundance (%) of each component of the essential oil was calculated according to individual and total peak areas. Various compounds were approved by their own standards [46].

### Method of determining the cytotoxic activity of the HPFEO

Ovarian cancer cell line (OVCAR-3) was prepared from Pasteur Institute of Iran (IPI) and cultured in 10 Roswell Park Memorial Institute (RPMI-1640) medium enriched with 10% fetal bovine serum (FBS), 2 mM L-glutamine and 1% anti-biotics (penicillin 100 U/mL and streptomycin 100 μg/mL) were incubated in an incubator at 37 °C under 5% CO_2_ pressure. After three passages, the cells were used for the next steps. MTT (3-(4,5-dimethylthiazolyl)-2,5-diphenyltetrazolium bromide) colorimetric method was used to evaluate the effect of essential oil on cell growth and proliferation. In summary, the suspension was first prepared from a cancer cell line and 20 µl of this suspension was added to each well from a 96-well plate; Each milliliter of culture medium contained 50,000 cells. After 24 h of incubation and assurance of cell adhesion to the plate bed, different concentrations of the HPFEO of 6.25, 12.50, 25, 50, 100, 200, and 400 μg/mL were added to each well. After 24 h of incubation, 20 μL of MTT dye at a concentration of 0.5 mg/mL was added to each well and the container containing the cells was incubated for 4 h. Then, the contents of the wells were carefully drained and replaced with a volume of 50 μL of dimethyl sulfoxide (DMSO). After 30 min of incubation and ensuring that the paint particles dissolved, the optical density of the treated samples or cells was calculated to be 570 nm. The concentration that inhibited cell growth up to 50% was considered as IC_50_. The experiment was repeated three times for the HPFEO. Cell survival percentage was calculated according to the following formula [47].

$$\mathrm{Cell\ viability}(\mathrm{\%}) = (\mathrm{OD\ treated}/\mathrm{OD\ control}) \times 100$$

### Method of determining the antimicrobial activity of the HPFEO

First, the standard microbial strains used in this study were prepared from the Scientific and Industrial Research Organization of Iran (IROST). Four strains were four Gram-positive bacteria (*Staphylococcus epidermidis* CIP 81.55, *Staphylococcus aureus* ATCC 29,737, *Streptococcus pyogenes* ATCC 19,615, and *Bacillus subtilis* ATCC 6633), five Gram-negative bacteria (*Klebsiella pneumoniae* ATCC 10,031, *Escherichia coli* ATCC 10,536, *Pseudomonas aeruginosa* ATCC 27,853, *Salmonella paratyphi A* serotype ATCC 5702, and *Shigella dysenteriae* PTCC 1188, and the two fungal strains of *Candida albicans* ATCC 10,231 and *Aspergillus brasiliensis* ATCC 16,404. To determine the zone of inhibition briefly using agar diffusion method, 100 μL of microbial suspensions (turbidity equivalent to half McFarland) were cultured on the culture medium. 10 μL of essential oil (30 mg/mL) was added to wells made on culture medium (6 mm in diameter). Then the plates related to bacteria were incubated for 24 h at 37 °C and the plates for yeast for 48 h and the plates for fungi were incubated for 30 h at 30 °C. The growth inhibition zone for each microorganism was determined by the antibiogram ruler in millimeters. To determine the minimum inhibitory concentration (MIC), in summary, each of the 96 house microplate wells had 95 μL of Brinhart infusion liquid culture medium, 5 μL of micro McFarland dilution suspension, and 100 μl of one of the HPFEO dilutions (4000, 2000, 1000, 500, 250, 125, 62.50 μg/mL) were added. Microplates inoculated with bacterial strains were then incubated at 37 °C for 24 h and yeast and fungal strains at 30 °C for 48 and 72 h. After leaving the greenhouse, the average of the first concentration that inhibited the growth of different strains was considered as the minimum concentration of growth inhibitor. 5 μL of each of the wells in which there was no growth was inoculated into nutrient agar medium and heated at 37 °C for 24 h. The mean of the first concentration that caused the killing of different strains was considered as the minimum concentration of microbial lethality (MBC/MFC) [48]. The experiment was repeated three times for the HPFEO.

### Statistical analysis

Statistical analysis was performed using Statistical Package for Social Sciences 22.0 software. Statistically significant evaluation of differences was performed using one-way ANOVA with 99% confidence level. The means were compared using Duncan's test with a probability level of one percent error.

## Results

### Content and components of the HPFEO

**Fig. 1:**
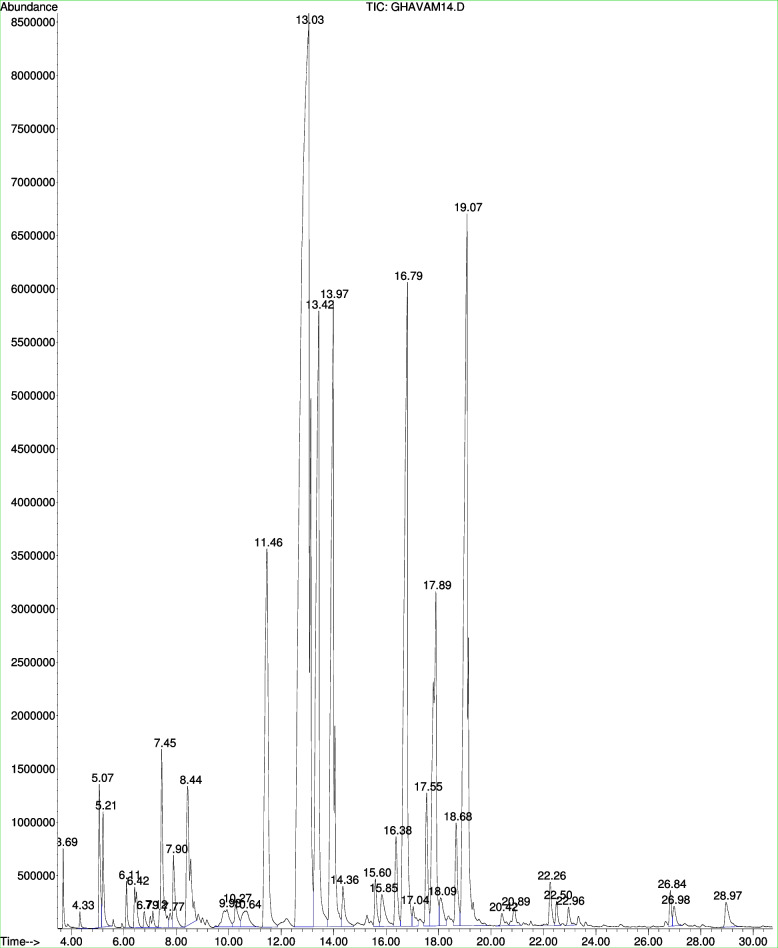
GC–MS chromatogram of the HPFEO

The HPFEO was white with a yield of 2.60 ± 0 0.00 w/w. The results of analysis by GC–MS of the HPFEO showed that there were 35 compounds with 99.54% relative content (Table 1 and Fig. 1). The ANOVA results showed that there was a significant difference between the mean amount of compounds obtained in the HPFEO (*P* ≤ 0.01).

**Table 1:** Chemical composition of the HPFEO

no	Compounds	RT	RI^c^	RI^s^	Content %	Molecular formula
1	Propanoic acid, 2-methyl-, 1-methylethyl ester	3.69	800.003	780	0.27 ± 0.00^o^	C_7_H_14_O_2_
2	Isopropyl butyrate	4.33	800.012	779	0.09 ± 0.00^r^	C_7_H_14_O_2_
3	Butanoic acid, 2-methyl-, 1-methylethyl ester	5.08	831.527	960	0.84 ± 0.00^j^	C_8_H_16_O_2_
4	Butanoic acid, 3-methyl-, 1-methylethyl ester	5.21	837.931	883	0.72 ± 0.00^ k^	C_8_H_16_O_2_
5	1R-α-Pinene	6.11	882.266	922	0.27 ± 0.00^o^	C_10_H_16_
6	Propanoic acid, 2-methyl-, hexyl ester	6.42	897.536	898	6.05 ± 0.00^e^	C_10_H_20_O_2_
7	2-Butenoic acid, 3-methyl, 1-methylethyl ester	6.79	900.662	940	0.14 ± 0.00^q^	C_8_H_14_O_2_
8	β-Pinene	7.12	921.523	970	0.19 ± 0.00^p^	C_10_H_16_
9	Butanoic acid, butyl ester	7.45	932,450	977	1.61 ± 0.00^ h^	C_8_H_16_O_2_
10	Octanal	7.77	943.046	987	0.13 ± 0.00^q^	C_8_H_16_O
11	Acetic acid, hexyl ester	7.90	947.666	995	0.71 ± 0.00^ k^	C_8_H_16_O_2_
12	D-Limonene	8.44	956.231	1020	2.06 ± 0.00^ g^	C_10_H_16_
13	Octanol	9.39	1011.640	1956	0.51 ± 0.00^ l^	C_8_H_18_O
14	Propanoic acid, hexyl ester	10.27	1020.634	1083	0.36 ± 0.00^n^	C_9_H_18_O_2_
15	Linalool	10.64	1030.423	1089	0.51 ± 0.00^ l^	C_10_H_18_O
16	Butanoic acid, hexyl ester = Hexyl butyrate	13.03	1093.650	1178	35.24 ± 0.00^a^	C_10_H_20_O_2_
17	Octyl acetate	13.42	1103.605	1194	8.42 ± 0.00^d^	C_10_H_20_O_2_
18	Butanoic acid, 2-methyl-, hexyl ester = Hexyl 2-methylbutyrate	13.97	1116.826	1240	8.14 ± 0.00^de^	C_11_H_22_O_2_
19	Pulegone	14.36	1126.201	1258	0.52 ± 0.00^ l^	C_10_H_16_O
20	Propanoic acid, octyl ester	15.60	1156.009	1284	0.46 ± 0.00^ lm^	C_11_H_22_O_2_
21	Anethole	15.65	1162.110	1285	0.60 + 0.26	C_10_H_12_O
22	Cyclohexane, ethenyl-	16.36	1174.759	918	0.96 + 1.06	C_8_H_14_
23	Propanoic acid, 2-methyl-, octyl ester = Octyl Isobutyrate	16.79	1184.615	1325	9.23 ± 0.00^c^	C_12_H_24_O_2_
24	3-Methyl-3-cyclohexen-1-one	17.04	1190.625	1197	0.25 ± 0.00^o^	C_7_H_10_O
25	2,4-Octadiene	17.55	1202.843	930	1.23 ± 0.00^i^	C_8_H_14_
26	Butanoic acid, octyl ester	17.99	1210.900	1372	5.01 ± 0.00^f^	C_12_H_24_O_2_
27	trans-3-Hexenoic Acid	18.09	1215.639	1021	0.47 ± 0.00^ lm^	C_6_H_10_O_2_
28	Butanoic acid, 2-methyl-, octyl ester = Octyl 2-methylbutyrate	19.07	1238.862	1421	11.65 ± 0.00^b^	C_13_H_26_O_2_
29	Germacrene D	20.42	1270.853	1481	0.18 ± 0.00^p^	C_15_H_24_
30	β-Bisabolene	20.90	1282.227	1503	0.27 ± 0.00^o^	C_15_H_24_
31	Germacrene B	22.26	1314.769	1526	0.47 ± 0.00^ lm^	C_15_H_24_
32	Hexanoic acid, octyl ester	22.50	1320.581	1570	0.21 ± 0.00^op^	C_14_H_28_O_2_
33	Butanoic acid, 3-methyl-, 3,7-dimethyl-2,6-octadienyl ester, (Z)-	22.96	1331.719	1582	0.19 ± 0.00^p^	C_15_H_26_O_2_
34	Octanoic acid, tetradecyl ester	26.84	1426.700	2377	0.29 ± 0.00^o^	C_22_H_44_O_2_
35	Estragole	28.97	1480.352	1198	0.43 ± 0.00^ m^	C_10_H_12_O
	Total				99.54	
	Monoterpenes hydrocarbons				2.52	
	Oxygenated monoterpenes				1.03	
	Sesquiterpenes hydrocarbons				0.92	
	Oxygenated sesquiterpenes				0	
	Others (nonterpenoids)				95.07	

RI^c^, linear retention indices on HP-5 column, experimentally determined using homologue series of n-alkanes (C8-C20). RI^s^, Linear retention index taken from Adams (2007), or NIST 14 (2014) and literature. Values with different letters are statistically different (Duncan, *p* ≤ 0.01); Mean (%) ± SD of three cultures were reported

Butanoic acid, hexyl ester (hexyl butyrate) with 35.24% was the dominant and main compound and butanoic acid, 2-methyl-, octyl ester (Octyl 2-methylbutyrate) was the second predominant compound in the HPFEO (11.65%). Propanoic acid, 2-methyl-, octyl ester (octyl isobutyrate) with the content of 9.23% was the third compound in the HPFEO. In the HPFEO, the fourth dominant compound was Octyl acetate with content of 8.42%.

### Cytotoxic activity of the HPFEO

**Fig. 2:**
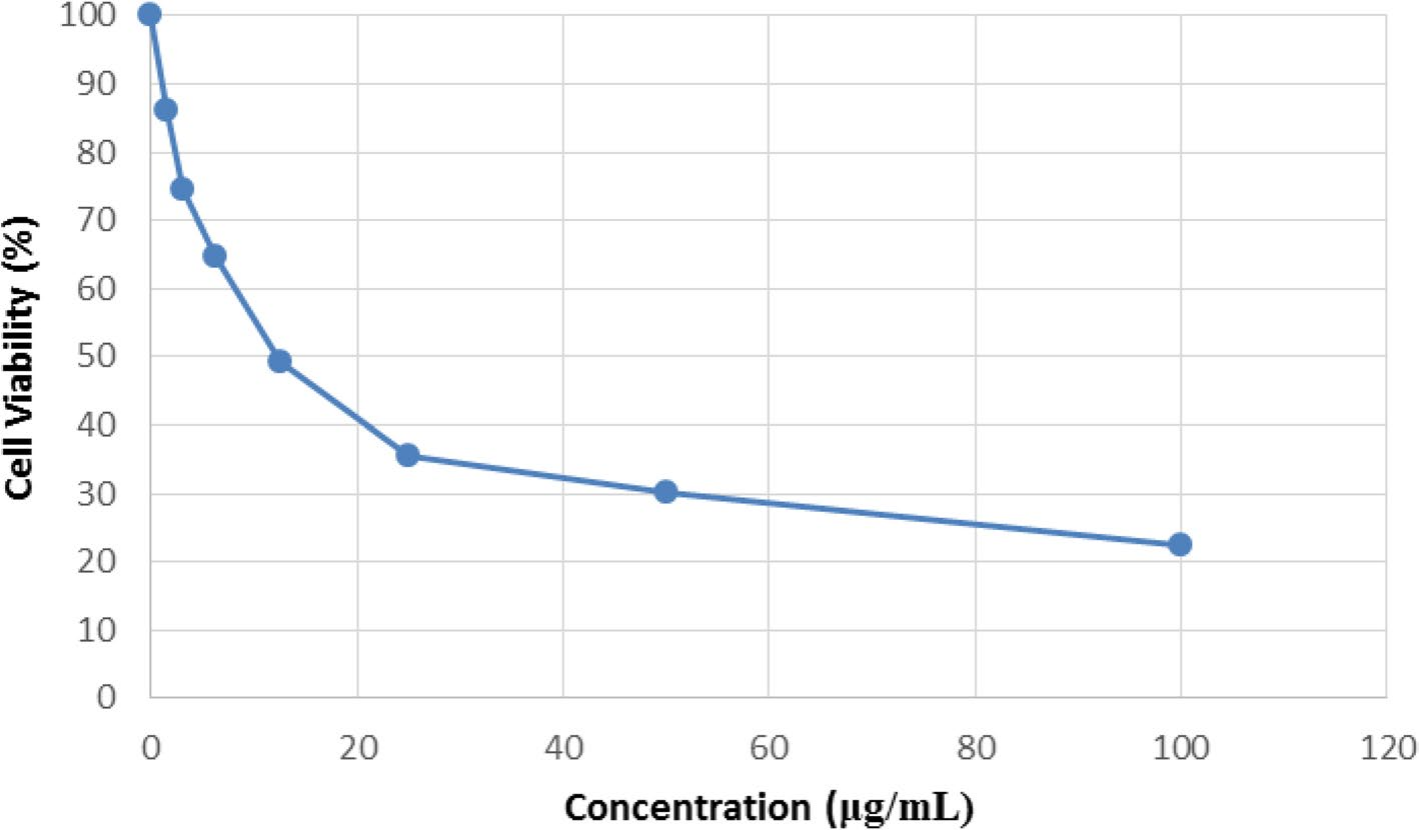
The dose-dependent cytotoxic effect on OVCAR-3 cell line of the HPFEO

Assessment of cell survival using MTT method on human ovarian cancer cell line (OVCAR-3) showed that the viability of these cells decreased in a concentration-dependent manner in 24 h (Fig. 2). Concentration of 1.56 μg/mL of the HPFEO showed a slight cytotoxic effect on this cell line. However, by increasing the concentration of the HPFEO from 3.12 μg/mL to 100 μg/mL, it increased the death of OVCAR-3 cells and reduced their survival in 24 h. The results showed that the HPFEO had value of IC_50_ 12.08 ± 0.06 μg/mL against OVCAR-3 cell line.

### Antimicrobial activity of the HPFEO

The results of antimicrobial activity from agar diffusion method indicated that the HPFEO oil did not create zone of inhibition against any of the studied microbial strains (Tables 2 and 3). The ANOVA results showed that there was a significant difference between the inhibitory and lethality activity of the HPFEO against different strains and positive controls (*P* ≤ 0.01). Based on the results of the minimum inhibition concentration (MIC), the strongest inhibitory activity of the HPFEO (MIC < 62.50 μg/mL) against Gram-negative bacterium *Pseudomonas aeruginosa* (ATCC 27,853), which was very strong and almost equal to rifampin (MIC = 31.25 μg/mL) had effected. However, it had a relatively good effect compared to gentamicin (MIC = 7.81 μg/mL).

**Table 2:** ZI, MIC, and MBC values of the HPFEO against the standard bacteria strains

standard bacteria strains	HPFEO			Rifampin	Gentamicin
	ZI (mm)	MIC ( μg/mL)	MBC( μg/mL)	MIC( μg/mL)	MIC( μg/mL)
*E. coli*	ND	< 62.50	< 62.50	3.90	3.90
*K. pneumoniae*	ND	500 ± 0.00^c^	1000	15.36	3.90
*P. aeruginosa*	ND	< 62.50	1000	31.25	7.81
*S. paratyphi-A*	ND	< 62.50	125	15.36	3.90
*Sh. dysenteriae*	ND	125 ± 0.00^c^	250	15.36	3.90
*B. subtilis*	ND	250 ± 0.00^c^	1000	31.25	3.90
*S. aureus*	ND	250 ± 0.00^c^	1000	31.25	1.95
*S. epidermidis*	ND	125 ± 0.00^c^	250	1.95	1.95
*S. pyogenes*	ND	< 62.50	< 62.50	0.975	0.975

*ND* not determined. Values with different letters are statistically different (Duncan, *p* ≤ 0.01)

**Table 3:** ZI, MIC, and MFC values of the HPFEO against the standard fungal strains

standard fungal strains	HPFEO			Nystatin
	ZI (mm)	MIC ( μg/mL)	MFC( μg/mL)	MIC ( μg/mL)
*A. brasiliensis*	ND	> 2000	> 2000	31.2
*A. sniger*	ND	> 2000	> 2000	31.2
*C. albicans*	ND	250 ± 0.05^b^	250	125

*ND * Not determined. Values with different letters are statistically different (Duncan, *p* ≤ 0.01)

Another significant inhibitory and lethal activity of the HPFEO was against *Salmonella paratyphi*-*A serotype* (ATCC 5702) (MIC < 62.50 μg/mL and MBC = 125 μg/mL) compared to rifampin (MIC = 15.36 μg/ mL) had a good effect and compared to gentamicin (MIC = 3.90 μg/mL) had a relatively weak effect against this bacterium. To date, there have been no reports of the effect of the HPFEO against *S. paratyphi-A*. *serotype*, and in this regard, our results are the first to suggest a potential natural potential against this bacterium.

The HPFEO also had a MIC and MBC value < 62.50 μg/mL against *Escherichia coli (*ATCC 25,922), which was three times weaker than the positive controls for rifampin and gentamicin (MIC = 3.90 μg/mL).

The activity of the HPFEO against Gram-positive bacteria *Bacillus subtilis* (ATCC 6633), *Staphylococcus aureus* (ATCC 29,737) in term of value MIC and MBC (MIC = 250 μg/mL and MBC = 1000 μg/mL) three times were weaker than rifampin (MIC = 31.25 μg/mL). On the other hand, the HPFEO with less inhibitory and lethal value (MIC = 125 μg/mL and MBC = 250 μg/mL) acted against the Gram-positive bacteria *Staphylococcus epidermidis* (CIP 81.55), however, compared to the positive controls of gentamicin and rifampin (MIC = 1.95 μg/mL) were 5 times weaker. No activity of the HPFEO against *S*. *epidermidis* has been reported so far.

Regarding the effect on Gram-positive bacteria, the lowest inhibitory and lethal value of the HPFEO was against *Streptococcus pyogenes* (ATCC 19,615) with a value of < 62.50 μg/mL. This activity was almost 5 times weaker compared to the positive controls of rifampin and gentamicin (MIC = 0.975 μg/mL). Since there has been no report of the effect of the HPFEO on the control and killing of this bacterium, our results can be significant and valuable for the production of a possible natural drug against this bacterium. Some components appear to be responsible for this activity.

One of the strong inhibitory and lethal activity of the HPFEO was against *Candida albicans* (ATCC 16,404) (MIC and MFC 250 μg/mL), which had a stronger functional effect compared to nystatin (MIC = 125 μg/mL).

The weakest activity inhibition and lethality of the HPFEO against *Aspergillus brasiliensis* (ATCC 16,404), *Aspergillus niger* (ATCC 9029 (> 2000 μg/mL), and Gram-negative bacteria *Klebsiella pneumonia* (ATCC 10,031) (MIC = 500 μg/mL and MBC = 1000 μg/mL). These differences may be due to differences in the chemical profile of the HPFEO in different regions and indicate a chemotype of this species.

## Discussion

The HPFEO was white with a yield of 2.60 ± 0 0.00 w/w which was in accordance with the results of [36] 2.5% in Yazd province and was the opposite with the results of [41] 3.8% in Mazandaran province, [32] 1.6% in Ardabil province, and [33] 1.6% in Kerman province. Essential oil yield is affected by the geographical conditions of the population. Environmental factors cause changes in the morphology and growth of medicinal plants as well as in the quantity and quality of active ingredients of essential oils [49].

Similarly, [32] reported 32 compounds with 99.86%, [17], 28 compounds with 97.18%. [39], with 45 compounds (99.92%) and [34] with 16 compounds (90.75%) identified the highest and lowest number of compounds in the HPFEO, respectively. Nonterpenoids were the major group of HPFEO compounds that is consistent with the results of previous studies on this essential oil [17, 32, 39].

Butanoic acid, hexyl ester (hexyl butyrate) with 35.24% was the dominant and main compound in the HPFEO, which according to the findings of 43.27%, 50.58%, 38.99%, 38.99%, 25.98%, and26.87% [17, 32–34, 37, 39]. Butanoic acid, 2-methyl-, octyl ester (octyl 2-methylbutyrate) was the second predominant compound in the HPFEO (11.65%), which was consistent with the results of [38] 14.2%. In the study of [37] and [35], this compound was reported as the third dominant compound with 14.37% and 4.2%, respectively. It has also been reported in many studies as a minor component [31–33]. The main reason for the difference between our results and previous research can be due to genetic factors, extraction methods, ecological conditions (climate, soil and geographical factors), environmental factors (light and temperature), plant growth stage, harvest season and storage conditions [39].

In the study of [37] this compound with the amount 17.82% and in the study of [38] with the amount of 8.2%, was reported as the second and fifth dominant composition of the HPFEO, respectively, which was not consistent with the present results. Also, in previous studies, octyl isobutyrate was mainly reported in the HPFEO, either unreported or in very small amounts (0.61–2.07%), which did not correspond to the present results [17, 33, 34]. Due to the differences of plants in different geographical areas that are collected in different seasons, a slight difference is predictable [38].

In the HPFEO, the fourth dominant compound was octyl acetate with content of 8.42%, which was consistent with the findings of [38] with content of 8.8%. Contrary to the present results, in many previous studies, this compound (9.8–27%) has been recorded as the second dominant compound of the HPFEO [17, 32–35]. On the other hand, the absence of this compound has been reported in some studies [36]. Butanoic acid, 2-methyl-, hexyl ester (hexyl 2-methylbutyrate) was in the next place with content of 8.14%, which was consistent with the findings of [34] with content of 4.27% as the fifth compound. A review of previous studies suggests that this compound was mostly either absent in plant fruit essential oil or has been observed in small amounts [31, 35]. Propanoic acid, 2-methyl-, hexyl ester (6.05%), Butanoic acid, octyl ester (5.01%) were other predominant compounds of the HPFEO. Similarly, these two compounds with amounts of 3.2 and 2.6% have been reported by [35]. Differences in the type and percentage of essential oils in different studies can be due to genetic or non-genetic changes in response to environmental differences in the habitat ecosystem [50].

According to official data from the World Health Organization, cancer is the second leading cause of death in the world's population and 9.6 million people died of the disease in 2018 [51]. Ovarian cancer, although it accounts for only 4% of all cancers in the female population, is the fifth leading cause of death from malignancy in women [52]. Plants have been shown to play a significant role in both the prevention and treatment of cancer [53, 54].

Similarly, [55] for ethanolic extract of *H*. *persicum* fruit. They reported cytotoxic activity against human lymphocytes and stated that cell viability was reduced by 100% at concentration of 10 μg/mL of extract. [38] reported inactive cytotoxicity of the HPFEO against HeLa (human cervical adenocarcinoma), LS180 (human colorectal adenocarcinoma), and Raji (human B lymphoma) cell lines with IC_50_ values ​​greater than 2000 μg/mL, which contradicts the present results. [34] reported cytotoxicity of the HPFEO using salinity test of saltwater shrimp (LC_50_ = 0.0071 μg/mL). Also, cytotoxic activity of *H. persicum* fruit extract was reported against breast cancer cells (MCF 7) (IC_50_ = 117.3 μg/mL) after 24 h, that these effects were due to the presence of furanocoumarin compounds in the extract of this plant and the effect on cell division process by interfering with the function of mitotic spindle microtubules [56].

The antitumor activity of the HPFEO with IC_50_ value of 2.24 mg/mL has been confirmed [40]. The biological effects of essential oils are directly related to the type of chemical compounds [57]. The predominance of aliphatic ester compounds such as hexyl butyrate, octyl 2-methylbutyrate, octyl isobutyrate, octyl acetate, hexyl 2-methylbutyrate, propanoic acid, 2-methyl-, hexyl ester, and butanoic acid, octyl ester seems to be the main factors on this activity. In a report, tumor inhibitory activity was attributed to hexyl butyrate and octyl acetate, which are the main constituents of the HPFEO [58]. These compounds act by different mechanisms, but the induction of apoptosis is a common point of many of these compounds. In addition, the minor compounds of essential oils can also affect the cytotoxic activity in synergy with the dominant compounds [59]. Therefore, the strong activity of the HPFEO can be related to the partial presence of monoterpenes such as β-pinene, D-limonene, linalool and pulegone and the susceptibility of terpenes such as germacrene D, β-bisabolene and germacrene B. The antimicrobial effects of pulegone and linalool compounds have been demonstrated [60]. The effect of germacrene D against human ovarian cancer cell line (OVCAR-3) has been reported [61]. The anti-proliferative effect of β-pinene against A-549 cell lines has been confirmed [62]. D-Limonene enhances the antitumor effect of dutaxel against prostate cancer cells without being toxic to prostate epithelial cells [63] The cytotoxic effect and apoptotic activity of anethole have been proven in various studies [64–66]. In this case, the essential oils are able to alter the polarity of cancer cells (especially mitochondrial membranes), ion channels, and disrupt membrane potential, thereby inhibiting the function of proton pumps and the production of ATP. Membrane ions may be degraded in the presence of plant essential oils, which leads to leakage of calcium ions and membrane proteins [67].

Essential oils prolong the delayed phase of bacterial growth and reduce the growth rate of bacteria in the logarithmic phase. Their antibacterial effect is due to their accumulation in two lipid layers of cell membrane and destruction of its structure [68]. Similarly, [40] for the HPFEO reported MIC value against *P. aeruginosa* was 12.5 ± 0.4 mg/mL, which had a very weak effect compared to the present study. The biological activity of essential oils depends on the type, nature and concentration of their compounds [69]. Therefore, it can be concluded that the biological effects of the HPFEO was related to its main components such as hexyl butyrate, octyl 2-methylbutyrate, octyl isobutyrate, octyl acetate. In addition, it seems that hydrocarbon monoterpenes such as D-limonene and oxygenated monoterpenes such as linalool and pulegone were other factors influencing this activity. The inhibitory effect of pulegone and D-limonene against *P. aeruginosa* has been reported [70]. The antibacterial effect of linalool against *P. aeruginosa* has been documented [71].

Similarly, [19] reported the inhibitory and lethal activity of the HPFEO against *Salmonella enterica* (MIC = 32 mg/mL and MBC = 64 mg/mL). On the other hand, the HPFEO against Gram-negative bacteria *Shigella dysenteriae* (PTCC 1188) has three times weaker inhibitory and lethal effect (MIC = 125 μg/mL and MBC = 250 μg/mL) compared to rifampin (MIC = 15.36 μg/mL) and four times weaker compared to gentamicin (MIC = 3.90 μg/mL). Since there have been no reports of the inhibitory and lethal effect of the HPFEO on this bacterium, our report can be valuable and significant. The effect of sesquiterpene on the inhibition of these strains has been confirmed [72]. Therefore, this activity can be related to the presence of germacrene D, β-bisabolene and germacrene B in the HPFEO.

Similarly, reports by [40] with MIC value of 25 mg/mL, [19] with value MIC 30 mg/mL and MBC 32 mg/mL have been recorded for the HPFEO, that contradicts by the present results. This different antibacterial activity in a variety of studies may be due to differences in growing location conditions, different essential oil extraction methods, or harvest time, leading to differences in essential oil composition and biological activity [73]. The presence of low terpene compounds as well as anethole seems to have been effective in inhibiting this bacterium. Effects of pulegone and D-limonene [70], germacrene D [74], linalool [71], α- and β-pinene [50, 75] and anethole [76] have been reported against *E. coli*.

[40] for the HPFEO against *S*. *aureus* and *B*. *subtilis* (MIC = 12.5 and 6.25 mg/ml) and [19] against *S*. *aureus* (MIC = 11 mg/mL and MBC = 16 mg/mL) reported antibacterial activity, which is contrary to the present results. The effect of α-pinene against *S*. *aureus* and *B*. *subtilis* has been reported [77] Linalool, limonene, pulegone, and anethole have been confirmed against *S. aureus* [70, 76].

The effect of essential oils containing monoterpenes such as α-pinene and linalool against this bacterium has been reported [39, 78].

Similarly, [38] for the HPFEO against *C. albicans* was reported zone of inhibition (6.4 ± 0.1 mm). Anethole seems to have been one of the main factors in its anti-yeast potency. [79] and [76] have reported strong ethenol activity against *C. albicans.* The anti-*C. albicans* effects of the monoterpenes such as limonene α-pinene, β-pinene, pulegone, and linalool have been reported [70, 80, 81].

## Conclusions

From the results of the present study, it can be seen that the importance of using the HPFEO in food and traditional Iranian medicine for the treatment of various diseases is due to its non-toxicity. The HPFEO was rich in aliphatic esters such as hexyl butyrate, octyl 2-methylbutyrate, octyl isobutyrate, octyl acetate, hexyl 2-methylbutyrate. The predominant compounds as well as the effect of monoterpene compounds and partial sesquiterpene caused the death of OVCAR-3 cells and reduced their survival in 24 h with IC_50_ value equal to 12.08 μg/mL. The HPFEO had a strong ability to inhibit Gram-negative bacteria such as *P. aeruginosa* and *S. paratyphi-A serotype* and the yeast *C. albicans*. This essential oil had a promising potential in inhibiting cancer cells and various microorganisms and requires future clinical research.

## Data Availability

The datasets used and/or analysed during the current study available from the corresponding author on reasonable request.

## Abbreviations

GC/MS: Gas chromatography–mass spectrometry
Μg: Microgram
mL: Miligram
MBC: Minimum bactericide concentration
MIC: Minimal inhibitory concentration
IROST: Iranian Research Organization on Science and Technology
MFC: Minimum fungicidal concentration
RI: Retention indices
